# Infographic: Slow-release dexamethasone in proliferative vitreoretinopathy (PVR)

**DOI:** 10.1038/s41433-021-01532-y

**Published:** 2021-06-02

**Authors:** I. Mostafa, M. Al-Zubaidy, A. Ghareeb, A. Song, A. Mehta, D. Murphy, S. Sadiq, N. Tzoumas, D. H. Steel

**Affiliations:** Institute of Biosciences, Newcastle University, Newcastle upon Tyne, United Kingdom

**Keywords:** Retinal diseases, Surgery

**Fig. 1:**
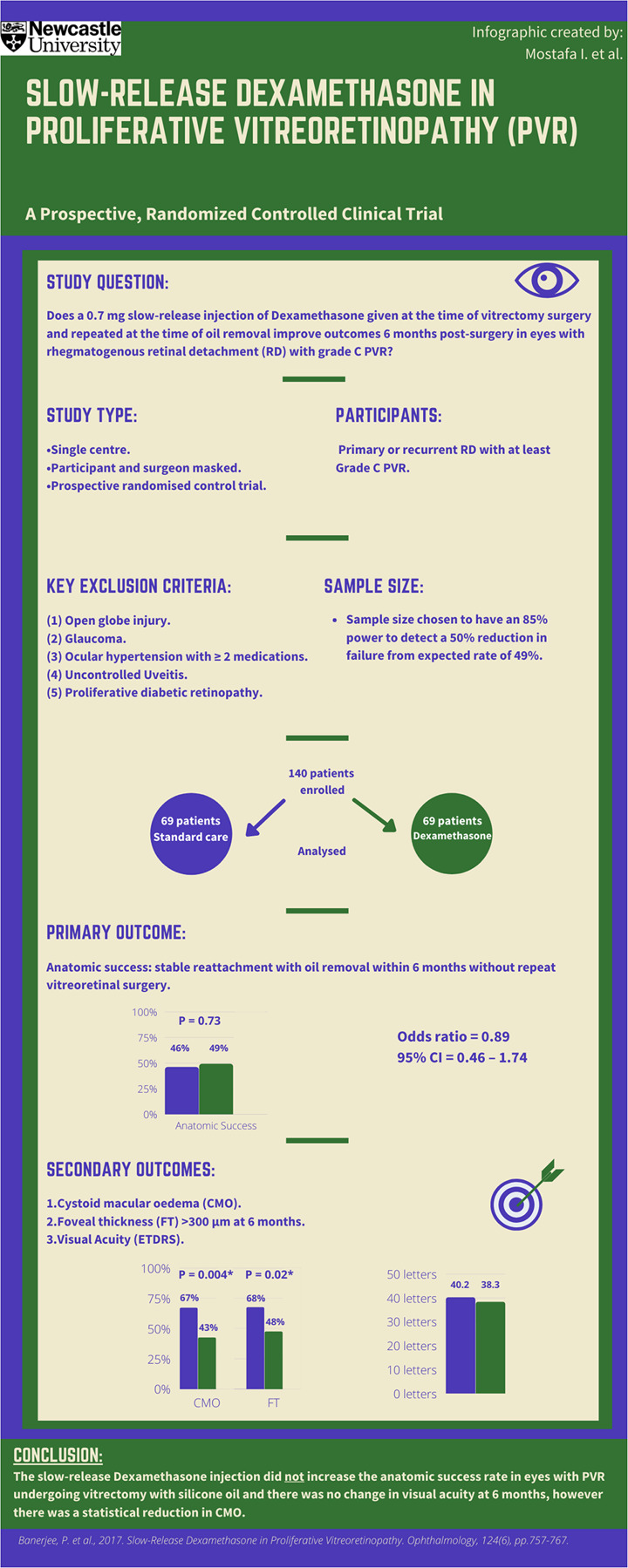
The slow-release dexamethasone in proliferative vitreoretinopathy (PVR) prospective, randomized controlled clinical trial showed that a slow-release Dexamethasone injection given at the time of vitrectomy surgery and repeated at the time of oil removal does not increase the anatomic success rate at 6 months in eyes with rhegmatogenous retinal detachment with grade C PVR. There was also no change in visual acuity, however there was a statistical reduction in cystoid macular oedema. CI confidence interval.

**Reference to original study:** Banerjee, P. et al., 2017. Slow-Release Dexamethasone in Proliferative Vitreoretinopathy. Ophthalmology, 124(6), pp.757–767.

